# Mobile Web-Based Monitoring and Coaching: Feasibility in Chronic Migraine

**DOI:** 10.2196/jmir.9.5.e38

**Published:** 2007-12-31

**Authors:** Marjolijn J Sorbi, Sander B Mak, Jan H Houtveen, Annet M Kleiboer, Lorenz JP van Doornen

**Affiliations:** ^2^Department of Information and Computing SciencesFaculty of Bèta SciencesUtrecht UniversityUtrechtThe Netherlands; ^1^Department of Clinical and Health PsychologyFaculty of Social and Behavioural SciencesUtrecht UniversityUtrechtThe Netherlands

**Keywords:** Personal digital assistant, ecological monitoring, electronics, migraine, health behavior, self-care, patient compliance, patient satisfaction

## Abstract

**Background:**

The Internet can facilitate diary monitoring (experience sampling, ecological momentary assessment) and behavioral coaching. Online digital assistance (ODA) is a generic tool for mobile Web-based use, intended as an adjuvant to face-to-face or Internet-based cognitive behavioral treatment. A current ODA application was designed to support home-based training of behavioral attack prevention in chronic migraine, focusing on the identification of attack precursors and the support of preventive health behaviour.

**Objective:**

The aim was to establish feasibility of the ODA approach in terms of technical problems and participant compliance, and ODA acceptability on the basis of ratings of user-friendliness, potential burden, and perceived support of the training for behavioral attack prevention in migraine.

**Methods:**

ODA combines mobile electronic diary monitoring with direct human online coaching of health behavior according to the information from the diary. The diary contains three parts covering the following: (1) migraine headache and medication use, (2) attack precursors, and (3) self-relaxation and other preventive behavior; in addition, menstruation (assessed in the evening diary) and disturbed sleep (assessed in the morning diary) is monitored. The pilot study consisted of two runs conducted with a total of five women with chronic migraine without aura. ODA was tested for 8.5 days (range 4-12 days) per participant. The first test run with three participants tested 4-5 diary prompts per day. The second run with another three participants (including one subject who participated in both runs) tested a reduced prompting scheme (2-3 prompts per day) and minor adaptations to the diary. Online coaching was executed twice daily on workdays.

**Results:**

ODA feasibility was established on the basis of acceptable data loss (1.2% due to the personal digital assistant; 5.6% due to failing Internet transmission) and good participant compliance (86.8% in the second run). Run 1 revealed some annoyance with the number of prompts per day. Overall ODA acceptability was evident by the positive participant responses concerning user-friendliness, absence of burden, and perceived support of migraine attack prevention. The software was adapted to further increase the flexibility of the application.

**Conclusions:**

ODA is feasible and well accepted. Tolerability is a sensitive issue, and the balance between benefit and burden must be considered with care. ODA offers a generic tool to combine mobile coaching with diary monitoring,independently of time and space. ODA effects on improvement of migraine remain to be established.

## Introduction

This paper presents a pilot study to test the feasibility and acceptability of a new method for mobile Web-based monitoring and coaching. Online digital assistance (ODA) was developed to monitor health and to coach health behavior in real life, independently of time or space, and as an adjuvant to cognitive behavioral treatment. ODA runs on advanced clinical software [1] and is executed through a personal digital assistant (PDA) or cellular phone with integrated Internet facility.

The monitoring feature of ODA is based on the electronic diary method of experience sampling [2,3] or ecological momentary assessment [4-6], employed in health psychology research [7-9] to reliably measure symptoms [10-12], momentary mood [13], or other fluctuating states in near real-time. PDAs are programmed to produce randomized calls, which prompt participants to answer diary items with the PDA keyboard or soft-touch screen. This generates valid real-time assessments of momentary health status and functioning, which are unbiased by anticipation or retrospection. The coaching feature of ODA is based on the possibility of connecting PDAs or cell phones wirelessly to the Internet, which permits tailoring feedback and behavior directives to users based on their information in the momentary diary. This requires specific software, called ODA 1.0, which was developed by students in the Utrecht University Department of Information and Computing Sciences [1].

ODA development was instigated within a Dutch trial on the effectiveness of a home-delivered behavioral training program conducted in 100 patients with chronic migraine [14,15]. Behavioral training in migraine is challenging [14] because the focus on attack precursors and preventive health behavior is counterintuitive to the attitudes and habits of many migraine patients. ODA monitoring could support the timely detection of attack precursors (the first behavioral training goal). This is essential because migraine precursors are elusive, and patients tend to focus on the agony of the attack and ignore its premonitory stage. ODA coaching could reinforce proactive behavior to prevent attack occurrence (the second behavioral training goal)*.*Behavior to slow down and sooth nervous system activation and brain stem disruption in the hours preceding the breakthrough of an attack is crucial, but it is contrary to the habit of many patients of increasing efforts and pushing toward finishing things in time before the pain strikes. Therefore, our first application of the ODA method pertained to supporting the identification of attack precursors and the execution of preventive health behavior in these patients.

We will present data on the feasibility and acceptability of this ODA application in five migraine patients [16,17]. ODA feasibility was assessed in terms of the technical problems encountered and participant compliance with the application. Technical problems are not expected to arise within the PDAs, which are quite robust. The ODA software and data transmission through the Internet could, however, encounter obstructions and thus induce data loss, which had to be evaluated. With respect to ODA participant compliance, we aimed to establish what proportion of diary prompts would be answered, which is a metric for compliance in experience sampling and ecological momentary assessment research [2-4].ODA acceptability was established on the basis of participant ratings of ODA user-friendliness, potential burden, and perceived support of behavioral attack prevention in migraine.

## Methods

### Participants

This study was conducted with five women with chronic migraine without aura [18]. The participants had successfully completed behavioral training and thus were well acquainted with its principles and aims. The first test run with three participants tested 4-5 prompts per day. The second run with another three participants (including one subject who participated in both runs) tested a reduced prompting scheme (2-3 prompts per day) and minor adaptations to the diary (Table 1). The Medical Ethics Review Committee of Utrecht University Medical Center approved the study.

**Table 1:** Characteristics of participants in the pilot study

**Participant**	1	2	3	4	5
**Run**	1	1	1 and 2	2	2
**Age (years)**	34	39	43	24	52
**Profession**	administrative employee	career advisor	industrial designer	psychologist	teacher
**Marital status**	married	single	single	married	married

### ODA Software, Monitoring, and Coaching

#### ODA Software

**Figure 1:**
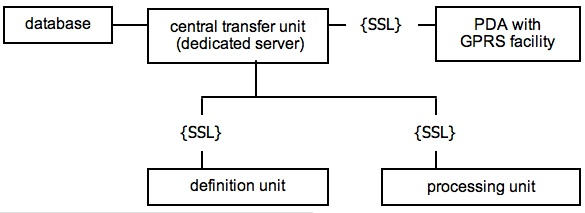
Design of software application ODA 1.0/1.1

A generic toolbox was built to allow adaptations to user characteristics and to the needs of researchers and clinicians. A convenient interface was promoted by keeping user actions to the utmost minimum and by facilitating the provision of online feedback. Privacy protection was assured by a dedicated server and Secure Sockets Layer (SSL), by authorization of users as well as researchers and clinicians through a personal log-in code and password, and by user-chosen nicknames. To avoid data loss, diary answers were separately saved on the server. The ODA software was extensively tested and reached its second milestone version [1]. It runs on Linux, supported by the software components Apache Web Server, Java, PostgresSQL, and Tomcat, and data encrypting is authorized by SSL certification. Figure 1 displays the units of the ODA software.

The definition unit serves to establish the design and setup of a given ODA application. This includes the documentation of demographics, diagnostics, and other particulars per user, such as type and dose of medication intake; the allocation of feedback providers (researchers or clinicians) to users; and the definition, handling, and storage of log-in codes, passwords, and user nicknames. Specifics of ODA monitoring are also defined here and include the diary editing, choice of answer categories per item, and the branching of items (meaning that the appearance of questions depends on the type of answer for a preceding question).

The processing unit handles the provision of the feedback for ODA coaching. It identifies the human feedback provider by log-in code and password, supplies Short Message Service (SMS) notification of completed diaries, and provides access to all current and archived user diaries. This unit also draws the attention of the feedback provider to extreme diary answers or patterns of scores by so-called alerts, which appear on a separate screen. The alerts underscore salient aspects of the diary, such as risk conditions or positive functioning, which are weighted against pre-programmed thresholds. Data are stored in ASCII files with a fixed matrix of variable names covering the time-stamped electronic diaries and feedback per user. These ASCII files are transformed through Excel for Windows (Microsoft Corporation, Redmond, WA, USA) into files suitable for data analysis in SPSS 14.0 (SPSS Inc, Chicago, IL, USA).

The PDA is pre-programmed with diary timer software developed at our institute and adapted to supplement the ODA software while optimizing the user-friendliness of the application [19]. The diary timer disables irrelevant PDA functions such as video recording and picture taking and locks the use of other PDA software. Primarily, however, it fine-tunes the electronic monitoring by creating and handling the prompting scheme. Pre-programmed definitions of this scheme involve the mean interval between calls, the variation range in random occurrence of the calls around these time points, and the desired interval (in minutes) between the repetitions of prompts per call. The diary timer also provides user options to regulate the volume of the prompting signal or chose a silent visual prompting mode for specific occasions (such as when attending a church service, meeting, or concert). It also provides a researcher option to limit the maximum duration of the silent prompting per day. Each day, the diary timer activates a randomized prompting scheme when the user enters a morning diary upon awakening, and it deactivates the prompting when the user enters an evening diary before going to sleep, which closely adapts the diary prompting to the individual wake-sleep cycle of the users. This function is new in electronic diary monitoring. It offers a strong advantage to user-friendliness, but it also weakens methodological control over the total number of calls per day, which depends on the duration of the user’s awake time. The diary timer produces a daily log file of the following: the times the user wakes up and goes to bed, all calls, number of prompts per call, and diary completion times.

#### Electronic Diary and Monitoring

**Figure 2:**
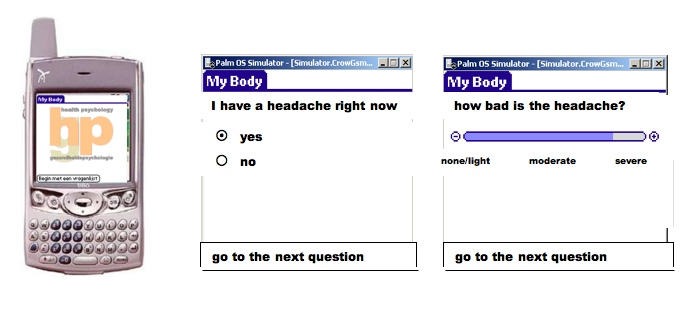
The PDA currently used for ODA and examples of diary items with response options

The ODA electronic diary is constructed according to the principles of experience sampling [3,6,11]: momentary functioning is captured by items that are short, unambiguous, grafted on the actual moment (right now), and that mimic the internal dialogue of the respondent through self-statements in colloquial language. Positively and negatively phrased items are balanced to avoid response tendencies, and answer categories include multiple choice, yes/no, and open answers, as well as visual analogue scales. Figure 2 shows the PDA (PalmOne Treo 600, Palm Inc, Sunnyvale, CA, USA) and examples of diary items as they appear on the PDA screen.

In experience sampling, a morning diary, an evening diary, and a day (beep) diary are employed. The user initiates a morning diary upon awakening to review the previous night and initiates an evening diary at bedtime to review the current day. Diaries during the day are prompted by randomized auditory calls or beeps. The frequency of this beep diary depends on the purpose of a given application. Usually, high-density monitoring with ≥ 10 beep diaries per day is confined to one to several days [20,21], while frequencies of four to six beep diaries per day may extend over weeks [10-12]. Responder compliance was satisfactory in applications of monitoring only [10-12,20], but in ODA several calls per day could be tolerated less well because the responder also receives ODA coaching and thus has additional tasks.

#### Coaching

The ODA software permits the provision of feedback that is fully automated [22]. The current focus is on the delivery of personal feedback that is closely tailored to the individual, thereby personalizing Web-based interventions to empower behavior change and self-care [23]. Feedback providers have exclusive access to the diary data of allocated participants and take care of all the feedback to those users. They remain anonymous, have no other contact (either direct, mail, or phone), and know the user exclusively by his/her nickname and diary entries.

**Figure 3:**
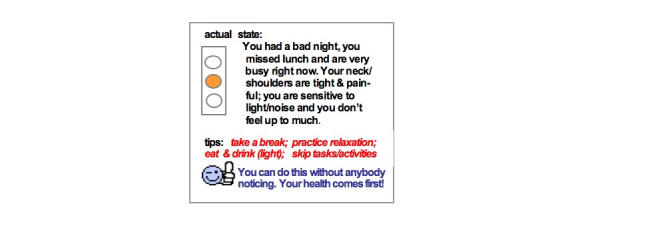
Example of the components of ODA feedback taken from the present ODA application in migraine

The feedback consists of three sections (in different colors on one page of the PDA screen) pertaining to the user’s actual state, tips for the user, and a pep-up statement. The software provides a second page to be scrolled through by the user, but this is rarely used. Feedback on the user’s actual state consists of a summary of salient information from the last diary regarding health risks or well-being accompanied by one of five color codes (green, green and orange; orange; orange and red; red) on a traffic light. Tips are behavioral directives for preventive self-care. Pep-up statements express encouragement, praise, support, or understanding of difficulties. They reinforce the execution of the tips and are underscored by an emoticon. Figure 3 provides an example.

ODA coaching is performed on laptop or desktop computers but can also be handled through a PDA. The evident advantage is that the feedback provider is mobile with the PDA, but its small screen hampers full inspection of the material to some extent. The feedback provider has access to each new diary and the ODA archive through log-in and password entrance to the ODA processing unit. Each new diary is accompanied by a record of alerts to gear the composition of the feedback. The current ODA application for migraine contains 17 alerts pertaining to healthy functioning, prevailing migraine headache or attack precursors, and the preventive health behavior employed by the user. Each alert is presented with its pre-programmed threshold and the diary scores involved. When composing the feedback, a written protocol of colors assigned to a 5-stepped hierarchy of health risks [16,17] dictates the choice of the traffic light, the emoticon is chosen from a programmed list of smileys, and the text is written as focused and condensed as possible. After checking the spelling and the match between text and graphics, the feedback is transmitted to the PDA of the user with an SMS notification that it is available.

### Procedures

#### Specifics of ODA Monitoring and Coaching

In run 1, subjects were signalled randomly in 2.5-hour time units, which represents a scheme of four to five calls per day, based on the compliance with comparable prompting in experience sampling studies in migraine [10], chronic pain [11,12,24], and severely fatigued subjects [25]. The prompts extended until 9:30 or 10:00 pm [10-12]or continued until bedtime [25]. Run 1 revealed annoyance with this number of calls per day, however. Annoyance would affect ODA tolerability and provided that the demands of behavioral training [14] limit the readiness of participants to invest extra effort, this would threaten the main study in which we intended to provide ODA while participants were undergoing behavioral training.

Therefore, a scheme of two to three calls per day was tested in run 2. In both runs, the option to put the prompting signal into silent mode was set at a maximum of 2 hours per day, and prompts were repeated three times per call with intervals of 1 minute. A beep diary not filled in after the third prompt was stored as a missed entry, and completely and partially entered diaries were automatically saved. Online coaching was confined to workdays and was provided twice per day by masters students in clinical and health psychology who were trained and supervised by the fourth author. For their convenience, they were allowed to incidentally provide feedback through a PDA.

#### Briefing and Debriefing

Participants were visited at home for 60-90 minutes of ODA demonstration and instruction and the signing of an informed consent form. They received a PDA with an instruction booklet and a phone number and email address to contact the researchers in case of questions or technical problems. The test run lasted for 8.5 days on average (range 4-12 days). After completion, the PDA was collected from the user’s home, ODA was evaluated, and the participant received a small gift.

### Instruments

#### Diary

The current 40-item beep diary contained three parts covering the following: (1) migraine headache and medication use, (2) attack precursors, and (3) self-relaxation and other preventive behavior. Items for part 1 were based on international diagnostic criteria [18] and adapted from items that had been successfully employed in previous studies [10-12]. Part 2 consisted of new items representing three classes of premonitory symptoms (eg, physical, affective-cognitive, behavioral) and triggers (eg, food intake, external strain, climatic conditions) of migraine attacks, derived from a literature review [16,17]. Items for part 3 were drawn from the behavioral training evaluation forms [14,15]. The 18-item evening and 19-item morning diaries included the items from part 1 of the beep diary as well as items for two additional migraine triggers: menstruation in women (assessed in the evening diary) and disturbed sleep (assessed in the morning diary).

#### Evaluative Questionnaire and Interview

The evaluation of ODA took place during the debriefing meeting at the user’s home. Participants filled in an evaluation form consisting of 11 items on user-friendliness, subjective compliance, the impact and potential burden of ODA, and the degree to which ODA supported the goals of behavioral training. The form included an open space for comments. This was complemented with a structured evaluative interview that focused on the peculiarities of handling the PDA, adhering to instructions, and managing eventual problems and on the positive and negative experiences with the system.

## Results

### Feasibility

#### Technical Problems in ODA

The loss of data due to technical problems amounted to 6.8% (11/161) potential diary entries. Minor internal problems occurred within the PDAs as expected and were solved by having the user reset the PDA. They accounted for a loss of 1.2% (n = 2) of the diary entries. External causes such as buildings or atmospheric influence incidentally hampered Internet transmission and thus receipt of the diary prompt by the user, which accounted for 5.6% (n = 9) of lost diaries. An initial software problem obstructed the storage of part of the feedback in run 1.

#### Compliance With ODA Monitoring

Table 2 summarizes the particulars of ODA monitoring and coaching separately for each participant and aggregated per run. In run 1, the mean compliance of 78.6% for the beep diary was just below the ≥ 80% criterion for good compliance, mainly due to participant 2 who missed almost half of the diary calls. The mean compliance in both runs was 80.1% for the beep diary, but in run 2, compliance with the beep diary was good (86.8%), as was the compliance in both runs with the morning and evening diaries.

**Table 2:** Objective compliance with ODA monitoring

	**Participant**						**Total Run 1**		**Participant**						**Total Run 2**	
	**1**		**2**		**3**				**3**		**4**		**5**			
	**No.**	**Mean or %**	**No.**	**Mean or %**	**No.**	**Mean or %**	**No.**	**Mean**	**No.**	**Mean or %**	**No.**	**Mean or %**	**No.**	**Mean or %**	**No.**	**Mean**
**ODA monitoring**																
Number of days	10		4		6		20	6.7	8		11		12		31	10.3
Number of diary calls^*^	41	4/day	16	4/day	30	5/day	87	4.3/day	21	2.6/day	25	2.3/day	28	2.3/day	74	2.4/day
Problems connecting to Internet	2		1		0		3		0		1		5		6	
Missed diary calls^†^	5	12.8%	7	46.7%	6	20%	18	21.4%	2	10%	5	16.7%	2	13%	9	13.2%
Completed beep diaries^†^	34	87.2%	8	53.3%	24	80%	66	78.6%	19	90%	19	83.3%	21	87%	59	86.8%
Completed morning diaries	8	80%	4	100%	6	100%	18	90%	7	88%	11	100%	7	58.3%	25	80.6%
Completed evening diaries	9	90%	4	100%	6	100%	19	95%	7	88%	9	81.8%	9	75%	25	80.6%
**ODA coaching**																
Number of workdays	6		4		4		14	4.7	6		7		8		21	7
Number of times feedback was given^‡^	9	1.5/day	3	0.8/day	7	1.8/day	19	1.4/day	12	2/day	14	2/day	16	2/day	42	2/day

^*^The number of calls per day depended on the setup (4-5 calls in run 1; 2-3 calls in run 2). The variation in calls received by the user depended on the time between wakeup and going to sleep, which differed between participants and between days per participant.

^†^Calls obstructed by Internet transmission problems were subtracted from the total number of calls in computing the percentages.

^‡^The days with feedback differed from the days of monitoring because feedback was not provided on the weekends, while monitoring continued. In run 1, an initial problem in data storage that was permanently solved accounted for smaller numbers than the feedback that had actually been provided.

### Acceptability

The findings from the 11-item ODA evaluation form are summarized in Table 3. Two of the three participants in run 1 objected to the number of diary calls per day. This was not the case in run 2, when the number of calls had been reduced. Since this was the only difference in the two runs, the remaining scores were averaged for the total group (last two columns of Table 3).

User-friendliness was rated positively regarding the handling of the PDA, readability of the screen, ease of answering diary questions, and clarity of the instructions. Perceived compliance with responding to the first prompt per call ranged from 50% to 75%. (Note that the computed compliance presented in Table 2 represents the response to all prompts per call.) The impact of ODA on daily living was consistently perceived as relatively low, as was the experienced burden (notwithstanding the dissatisfaction with the number of calls in run 1). According to the participants, ODA supported the key targets of behavioral training to a considerable extent, and 80% were ready to participate again.

**Table 3:** Perceived user-friendliness, compliance, impact, and behavioral training support of ODA

	Run 1^*^ (N = 3)		Run 2^*^ (N = 3)		**Run 1 and 2(N = 6)**	
	**Mean**	**Range**	**Mean**	**Range**	**Mean**	**Range**
**User-friendliness of the PDA**^**†**^						
Did you have trouble handling the PDA?	1.7	1-3	1.0	1	1.4	1-3
Did you have difficulties reading the words on the PDA screen?	1.7	1-3	1.0	1	1.4	1-3
Could you conveniently answer the diary on the PDA?	3.7	3-4	4.0	4	3.9	3-4
Were the instructions clear to you?	3.3	2-4	4.0	4	3.7	2-4
**Immediate compliance with online monitoring**^‡^						
How often did you fill in the diary directly after the first prompt?	2.7	2-3	2.0	1-4	2.3	1-4
**Impact of ODA**^†^						
Did you experience your participation as a burden?	2.3	2-3	2.0	2	2.2	2-3
Did the study influence your daily life?	2	1-3	2.3	2-3	2.2	1-3
Was the number of calls per day annoying?	3.3	2-4	1.3	1-2		
Would you agree to participate again in a comparable study?	4	4	3.0	2-4	3.5	2-4
**ODA support ofbehavioral training key targets**^**†**^						
Did the diary help detect early symptoms and migraine triggers?	2.7	2-3	3.3	3-4	3	2-4
Did the feedback help you to take actions to prevent an attack?	3	3	2.7	2-3	2.9	2-3

^*^Run 1: 4-5 calls/day; run 2: 2-3 calls/day.

^†^1 = no/not; 2 = somewhat; 3 = considerably; 4 = very much.

^‡^1 = in less than 50% of the beep diaries; 2 = in approximately 50%; 3 = in approximately 75%; 4 = in almost all beep diaries.

The participants’ comments and responses in the evaluative interview underscored that ODA was confronting in the sense that it induced awareness of the user’s denial of premonitory symptoms and then counteracted this neglect. ODA was also said to have induced timely and more frequent self-relaxation, pacing down, and careful attention to actual personal needs. ODA coaching was experienced as an essential incentive that made the difference in actually taking measures against overexertion. Interestingly, the existence of an external voice or person was regarded as decisive in this matter. ODA was not automatically or directly accepted—it required time for the users to become acquainted with and get used to. When this was accomplished, the PDA providing ODA was experienced as a supportive little helper or companion, conveying that one is not left alone and that migraine self-management is a shared problem. Consequently, some of the participants asserted to have missed the feedback on the weekends, and one participant dreaded handing in the PDA.

## Discussion

This study showed that ODA successfully produced electronic diaries of momentary state and functioning (provided online by users) as well as direct coaching of health behavior (provided online to users by trained research assistants). Data loss due to internal causes was negligible (1.2%) and was acceptable when due to external causes (5.6%). Compliance with ODA monitoring was > 80% (86.8% in run 2), user-friendliness was confirmed, and the perceived burden was low. ODA did not disturb normal life to any considerable degree and was well accepted and appreciated by the participants.

We underscore that ODA was developed not to refine experience sampling methodology, per se, but to provide a mobile tool for online monitoring and coaching as an adjuvant to face-to-face or Internet-based cognitive behavioral treatment. Below we discuss issues in ODA development, technical problems, and potential data loss and issues of ODA tolerability.

ODA development was not an easy venture. Part of the difficulty arose from the fact that the sales information of PDA-producing companies insufficiently covered areas of internal functioning, which were specifically relevant to applications such as ODA. As far as we can see, this holds for all PDAs on the market and applies also to cell phones. The PDA Internet facility, in particular, had flaws obstructing the development of ODA. Problems popped up unexpectedly irrespective of the degree of software testing, and in our experience, these could be solved exclusively through Internet communication with other users of the hardware at issue. These problems were handled before the execution of the present pilot study. Within this study, the performance of ODA encountered few PDA-intrinsic problems after the devices were handed out to the users (1.2%).

An important source of data loss to be considered was Internet transmission problems due to external conditions such as buildings or atmospheric influence. These external causes induced a data loss of 5.6% in the present study, which was considered acceptable. The flaw was not due to ODA software or hardware or to problems of users. There were other external sources of potential data loss as well. We encountered an impending disturbance in the receipt of the feedback due to irregularities induced by the SMS provider, and the server was blocked for several hours because of unannounced maintenance work. Fortunately, both events did not induce actual data loss. These underscore, however, that services provided by external agencies for SMS delivery or server hosting demand watchful attention.

Tolerability and acceptability of ODA protect against data loss due to non-adherence. Tolerability is a particularly sensitive issue when ODA is used to support cognitive behavioral treatment because burdening or annoying participants could hamper instead of promote progress of the intervention. Migraine sufferers had accepted six prompts for more than 2 months of electronic diary keeping [10], and subjects with chronic pain [11,12] or fatigue [25] had tolerated four to five prompts per day for 2 to 4 weeks. Two of the three participants objected to this number of prompts in run 1 of the present pilot study. This finding may be coincidental, given the small number of subjects. We took it seriously, however, because ODA can be demanding, and careful attention should be devoted to prevent the accumulation of burden.

We present four potential threats to ODA tolerability. First, users may be bored if diary items are not presented flexibly through branching logics. We took this risk in part 2 of the present diary where bulky sets of items were required to cover potential premonitory symptoms of the migraine attack. All of these potential symptoms had to be explored in each diary because, thus far, they were poorly understood in the empirical literature. Second, in the present ODA application, diary answers were saved separately to the database to guard against data loss. As a consequence, respondents had to wait briefly after each answer, and the average diary completion time was 8 minutes instead of 5 minutes in previous studies. The present findings show that data loss was limited in ODA. We therefore recommend against separate answer transmission through the Internet because the advantage of speed and fluency of the diary completion seems to outweigh the gain of preventing extra data loss. Third, in ODA, diary monitoring is not the only task. The feedback from online coaching also requires attention, and the feedback was, at times, experienced by the user as confronting and thus might have been energy consuming. This implies that ODA coaching could be burdening in its own right. Fourth, it deserves considering that PDAs might have been accepted particularly well in early experience sampling studies because at that time they were a novelty gadget [10]. At present, however, interruptions from mobile devices are burdening [26] and meet zero tolerance in many circumstances. Therefore, this might also threaten the tolerance of ODA and electronic monitoring in general.

These issues underscore that the combination of online monitoring and coaching involves participants more strongly than monitoring alone did in previous studies and that ODA requires a careful account of the balance between benefit and burden. All the factors mentioned here could affect ODA tolerance, which could, in turn, be reinforced by the demands of being under treatment. In order to optimize ODA tolerance, we took measures to increase ODA flexibility based on the present findings. The ODA software was accustomed to saving diaries on the server upon completion, which produces a 40% reduction in the completion time of equivalents of the current diary, and the diary timer software was adopted to restrict calls to certain times of day or to exclude prompting in the evening when desired while keeping the adaptation to the individual wake-sleep cycle of the users. New steps in technology development concern full implementation of ODA in cell phones and extension of the ODA software to create individualized graphical representations of selected diary scores, which provides users with fully automated feedback on the course of their health status over time. Currently, we are linking ODA to Web portals and other eHealth applications. One possibility concerns connection to a German system for computer-aided therapy [27] that shares the same software basis.

We conclude that we succeeded in developing a new tool for mobile Web-based monitoring and coaching. ODA feasibility and acceptability are attainable, and ODA can be used conveniently to assess momentary functioning and to deliver direct feedback and behavioral directives while reaching people independently of time and space. ODA fulfilled its specific promise to the current application in migraine: reinforcing risk detection and behavioral attack prevention, the central goals of behavioral training in migraine. We underscore that the present findings can not be generalized to other populations that might be less motivated or show higher attrition rates. Large-scale use of ODA is not warranted as yet since ODA effectiveness remains to be established. This is at issue in a current study of the ODA application in migraine.

ODA could easily be extended to other areas using shorter diaries and the inclusion of fully automated feedback. A potentially promising field is that of health maintenance and optimal functioning in corporate organizations. The present paper may encourage efforts in other fields, particularly areas in which lifestyle change prevails and mobile contact is practical. ODA intended as a supplement to Internet-based cognitive behavioral intervention may also have potential in its own right as a tool in research or clinical practice to support prevention and health self-management.

Last, we point out that participant comments in the current ODA feasibility study hint at intriguing new issues in eHealth. The PDA was experienced as a personal companion and helper with a voice. This experience was crucial in instigating action, actual goal-directed behavior, and preventive self-care. Could it be that mobile digital devices come closer to the individual and have greater potential to actually change health behavior than current media-transmitted health education and prevention programs? This is a challenging question that, in our view, deserves serious research attention.

## Abbreviations

behavioral training: behavioral training
ODA: online digital assistance
PDA: personal digital assistant
SSL: Secure Sockets Layer
SMS: Short Message Service
